# The evaluation of latent fingerprints exposed to different snow conditions and their usability in forensics

**DOI:** 10.1093/fsr/owaf019

**Published:** 2025-07-29

**Authors:** Michal Soták, Mária Chovancová (nee Kondeková), Petra Švábová (nee Uhrová), Radoslav Beňuš

**Affiliations:** Department of Anthropology, Faculty of Natural Sciences, Comenius University of Bratislava Mlynská Dolina, Bratislava, Slovak Republic; Department of Anthropology, Faculty of Natural Sciences, Comenius University of Bratislava Mlynská Dolina, Bratislava, Slovak Republic; Department of Anthropology, Faculty of Natural Sciences, Comenius University of Bratislava Mlynská Dolina, Bratislava, Slovak Republic; Department of Anthropology, Faculty of Natural Sciences, Comenius University of Bratislava Mlynská Dolina, Bratislava, Slovak Republic

**Keywords:** forensic sciences, personal identification, dermatoglyphics, latent fingerprints, degradation, environmental factors, snow conditions, forensic anthropology, usability

## Abstract

Second level dactyloscopic markants (minutiae) are irregularities in the course of the friction ridge skin used for personal identification because of their high variability. Individual uniqueness is affected by the high number of minutiae and their random distribution within the friction ridge skin. The combination of various environmental factors, e.g., snow, soil environment, and direct sunlight, can affect the quality of fingerprints. The aim of this study was to analyze the influence of snow under two different conditions (natural snowfall and immersion in the snow) within various time intervals on fingerprints. A total of 265 groomed latent distal fingerprints were taken from a Caucasian male from Slovakia. Latent fingerprints were taken only from one participant, as the composition of sweat, i.e., the sweat-fat substance, varies from person to person, which could influence the results. Subsequently, obtained latent fingerprints were developed using small particle reagent. We evaluated the decrease in the number of minutiae on latent fingerprints exposed to the destructive influence of snow. The results confirmed that snow has a significant effect on the quality of latent fingerprints, as a decrease in the average number of minutiae on latent prints was observed after only 2 h of exposure to snow conditions. After snow exposure, usable fingerprints for identification decreased, whilst non-usable ones increased, with 53.33% of non-usable fingerprints resulting from natural snowfall exposure. After exposure of latent fingerprints to snow immersion, 98.13% of non-usable fingerprints were found. The results can help improving personal identification efficiency.

**Key Points**
 

- Fingerprints exposed to various environmental factors are recommended to be evaluated.
- Visual quality of latent prints decreases with the length of exposure to snow conditions.
- The average number of minutiae decreases significantly after the influence of snow conditions.
- Snowfall has a less negative influence on the average number of minutiae than forcible immersion.
- Latent fingerprints exposed to snowfall have better identification potential.

## Introduction

Forensic science is used to analyze evidence from crime scenes to establish a direct connection to offenders and support their personal identification [1]. A key focus is the collection of biological evidence, with dactyloscopy being a relevant method [2]. Dactyloscopy involves the analysis and classification of the friction ridge skin characteristics observed in fingerprint impressions, which are unique to each individual. The basic characteristics of fingerprints are durability, immutability and individuality. It involves collecting friction ridge skin impressions from fingers, palms, and soles to support crime detection, investigation, and prevention [3–6].

Fingerprints are one of the most valuable pieces of evidence found by forensic experts on various objects at crime scenes used for personal identification [7, 8]. Fingerprints are found at the crime scene in various forms—patent, plastic, and latent [9]. Patent fingerprints are simply visible and need only be preserved. They can be found in dust, ink, or blood. Latent prints are invisible to naked eyes and can only be revealed using physical or chemical processes designed to enhance the fingerprint left by the sweat-fatty substance of the friction ridge skin [10]. A plastic fingerprints form when friction ridge skin press into a soft surface, creating three-dimensional impression. Such prints can appear in materials like wax, putty, or melted plastic [9, 11].

Forensic experts often recover only partial fingerprints or palm prints [12]. Latent fingerprints consist of sweat secretions and fatty components. Eccrine glands, which serve a thermoregulatory function, secrete water-based sweat without cellular debris through pores on the friction ridge skin, leaving behind prints upon contact with surface [13, 14].

Latent fingerprints are divided into two categories—groomed and natural. Groomed latent fingerprints are formed when individuals rub their own fingertips on their oily facial or neck skin, enriching the prints with sebum. Natural latent fingerprints consist of the residue naturally present on the fingertips when the hands have not been intentionally washed or cleaned before leaving latent fingerprints [15, 16].

On human fingertips, we observe identification features of three levels. First-level features are pattern types such as arch, loop, whirl, and complicated patterns. These are transferred to the fingerprints and are used mostly to observe population variability [6, 17].

Second-level features are minutiae, which are defined as irregularities in the course of the friction ridge skin. The individual uniqueness of each fingerprint is influenced by the high number of minutiae and their random distribution within the friction ridge skin [8, 18]. The distribution of identifying features on fingerprints has sufficient discriminatory power to determine an individual’s identity [18]. The distribution of second-level features on fingerprints is unique to each person [6, 8]. The names of the individual features are most often derived from their shapes (e.g., dot, bifurcation, bridge, and overlap) [19]. In Slovak dermatoglyphic and forensic practice, the classification according to Gutiérrez et al. [8] is used, which characterizes 13 types of minutiae.

Third-level identification features serve as additional characteristics that can confirm or deny a match in personal identification, including scars, sweat pores, creases, or incipient ridges [5, 17, 18].

In personal identification, not only the types of minutiae are important, but also their frequency [8]. Fingerprints in personal identification are classified as: usable, partially usable, or non-usable. In Slovak forensic practice, a fingerprint with at least 10 minutiae is required for personal identification [20]. There is no clear justification for the necessary number of minutiae set for the conclusion on the fingerprint matching. It is based on the experience in probability, estimation and the experience of the investigators. According to internal regulations, at least 10 minutiae are required to consider a fingerprint usable. Fingerprints with 7–9 minutiae are partially-usable, and those with fewer than seven are non-usable [4]. Fingerprint quality may be reduced not only by partial fingerprints but also by environmental factors at the crime scene, which remain relatively under-researched [21].

Environmental factors significantly affect the quality of latent fingerprints [22]. Their combination can even completely disrupt the friction ridge skin of the print [7]. Although this influence is not yet fully studied, thorough investigation could help develop systematic methods applicable in forensics [23]. In particular, researchers focused on whether it is possible to develop fingerprints after exposure to various environmental factors, but not on the impact on second-level features [24]. The only known study observing the impact of environmental factors on minutiae was conducted by De Alcaraz-Fossoul et al. [25], who concluded that degradation effects are highly dependent on the interaction of environmental factors and minutiae count. In addition, fingerprints are exposed to different environmental factors such as snow [22], direct sunlight [7], soil [26], and water [27].

Some studies confirm the influence of environmental factors on the quality of latent fingerprints. McCook et al. [22] evaluated latent fingerprints left in freezing environments over time. After the first week, the quality of the latent fingerprints was very high. After the second and third weeks, the quality of latent fingerprints was non-significantly lower than that after the first week.

The water environment also has a destructive effect on latent fingerprint quality. In a study by Trapecar [28], the quality of latent fingerprints was observed for 15 days. Due to the large number of degraded latent fingerprints stored in a water environment over this period, the number of degraded latent fingerprints is expected to increase as a function of time [29].

The combination of light and heat has a significant effect on the visual degradation of latent fingerprints. Each combination of factors affects the level of chemical and visual degradation differently, making it harder to determine the time of fingerprint deposition. Sunlight alone has less impact than when combined with elevated temperatures. If latent fingerprints are in a light, warm and less humid environment, the level of degradation is lower. Higher humidity increases visual degradation. Non-porous surfaces better preserve fingerprints exposed to sunlight. Although sweat evaporates from latent fingerprints on non-porous surfaces, the degradation of these fingerprints is less severe compared to those left on porous surfaces [7].

Latent fingerprints exposed to snow should be secured despite its destructive effect [30]. Most commonly, latent fingerprints are found in cases where criminals embed various objects (e.g., weapons) in the snow in an attempt to hide their fingerprints [31].

Natural snowfall is an environmental factor that adversely affects the quality of latent fingerprints, even though some minutiae may be retained [22]. The duration of exposure to an unprotected winter environment and the method of embedding the object in the snow can impact the quality of latent fingerprints [30]. The mechanical force exerted when immersing an object in snow has more destructive effects than snowfall alone, significantly reducing the number of identifiable minutiae [22].

Another factor that can affect the quality of latent fingerprints is the choice of the development method and the correct technique for developing latent fingerprints. The quality of latent fingerprints can be particularly influenced in cases where they are exposed to environmental factors [21, 32].

Small particle reagent (SPR) developer is an effective method for developing latent fingerprints on wet and non-porous surfaces [33]. The reagent contains molybdenum disulphide in the surfactant solution, which can be replaced in the solution by titanium dioxide, zinc oxide, magnetite, graphite, or zinc carbonate [14, 34]. Fluorescent compounds (violet crystal, rhodamine, and others) are added to produce small particle fluorescent reagents [34, 35]. Unlike powders requiring dry surfaces, SPR adheres to fatty components of latent fingerprints on wet surfaces [14]. Before use, the reagent is mixed and then it is sprayed onto the wet surface [36]. The surface should be allowed to dry, and the developed fingerprints should be secured using dactyloscopic tape or foil [34].

This pilot study examines whether latent fingerprints exposed to snow conditions can still be developed and used for personal identification. It evaluates the impact of natural snowfall and forced immersions into snow on fingerprint quality, particularly regarding second-level features (minutiae). The findings may support forensic efforts in recovering fingerprints from snow-affected crime scenes.

## Materials and methods

A total of 265 groomed latent distal fingerprints were taken from a Caucasian male from Slovakia with written informed consent form. Latent fingerprints were taken only from one participant, as the composition of sweat, i.e., the sweat-fat substance, varies from person to person, which could influence the results. The fingerprints obtained were subsequently exposed to snow conditions in two ways: natural snowfall and the forced immersion of dactyloscopic glass plates with latent fingerprints into the snow for specific time intervals. The data collection was a part of a dermatoglyphic research project and the research was approved by the Ethics Committee for Human Research of the Faculty of Natural Sciences, Comenius University, Bratislava, Slovakia (Approval No. ECH19019).

The dactyloscopic glass plates were cleaned with clean water before use to prevent contamination and remove any dirt. Groomed latent fingerprints were then placed on the glass plates and exposed to two snow conditions for 2 h to 2 d. The first set of plates with 105 latent fingerprints was exposed to the effects of natural snowfall, whilst the second set of 160 latent fingerprints was exposed to the effects of forced immersion in the snow. The forced immersion in the snow occurred at approximately the same angle and with approximately the same force. The dactyloscopic glasses were inserted into the snow from an identical height, in the same motion to the same depth, so that the upper edge of the dactyloscopic glass was 5 cm below the snow surface. When observing the effect of snow conditions, control samples were also evaluated for both effects of snow conditions. The control sample was developed and evaluated as soon as the fingerprints were left on the dactyloscopic glass. The control sample of latent fingerprints served as our comparison group in evaluating differences in the average number of minutiae on degraded latent fingerprints. The temperature of the environment in which the latent fingerprints were exposed was measured during exposure to the environmental factors (Table 1) using a ThermoPro TP357 room thermometer (ThermoPro Inc., Toronto, Ontario, Canada).

**Table 1:** Mean ambient temperature during latent fingerprint exposure to natural snowfall and forced snow immersion.

Factor	Time interval	Average temperature (°C)
Natural snowfall	2 h	−2.50
	12 h	−2.25
	1 d	−1.79
Snow (forced immersion)	2 h	0.50
	12 h	−1.83
	1 d	−1.92
	2 d	−1.80

Note: The temperature of 0.50°C reflects the actual environmental conditions during fingerprint exposure.

In previously conducted studies, fingerprints were left on a variety of non-porous materials (e.g., cans, ceramic tiles, metal spoon, aluminium foil) [15, 17]. Dactyloscopic glass plates were selected as the non-porous material to accommodate larger latent fingerprint samples. Unlike McCook et al.’s [22] controlled conditions in a freezer, this pilot study exposed latent fingerprints to uncontrolled outdoor conditions, necessitating a modified methodology.

### Development and evaluation of degraded latent fingerprints

At each time interval (2 h, 12 h, 1 d, and 2 d), the glass plates with latent fingerprints were brought indoors where the snow was allowed to melt naturally. After the snow had melted, the latent fingerprints were developed using SPR black. The glass plates with developed latent fingerprints were allowed to dry. Developed fingerprints were removed by adhesive tape, which was always pressed with sufficient force on the developed fingerprint. The tape, along with the developed fingerprint, was slowly peeled off from the glass plate and transferred onto paper.

To develop control sample fingerprints, we used a magnetic silver–grey Hi-fi dactyloscopic powder (LT Sezam s.r.o). This powder was applied with a magnetic dactyloscopic brush MaxMag (15 cm) (LT Sezam s.r.o), which is a magnetic brush with a strong magnet, for working with larger amounts of powder.

**Figure 1:**
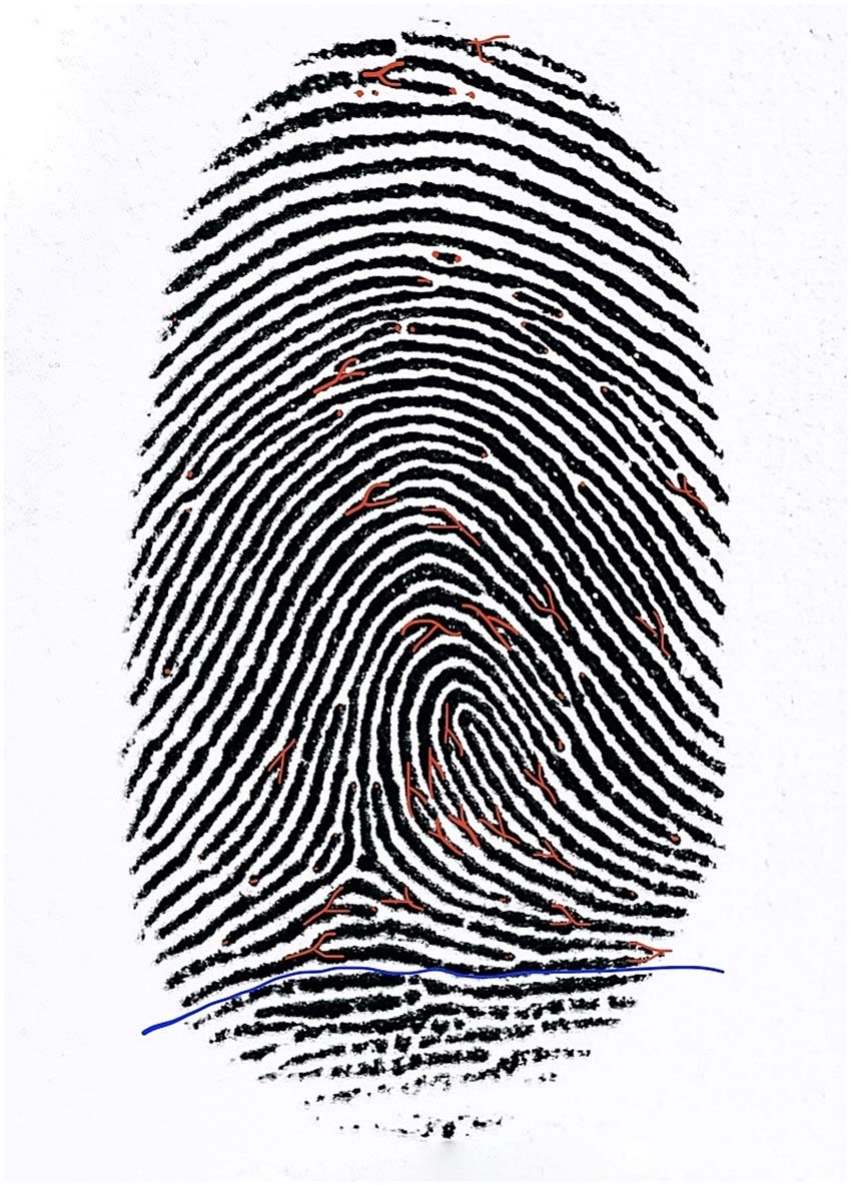
The evaluated minutiae on a fingerprint template.

Developed latent fingerprints were scanned at a resolution of 1 200 dpi using a Brother DCP-L2512D printer (Brother Industries, Ltd., Nagoya, Japan) and then evaluated in Preview 11.0 (1048) by Apple Inc. Minutiae counts were compared between control and degraded fingerprints at each time interval. To avoid error and missed minutiae, a fingerprint template was used to evaluate the number of minutiae on control sample and degraded fingerprints (Figure 1). The minutiae on degraded fingerprints were evaluated using commonly used established methods by Gutiérrez et al. [8].

**Figure 2:**
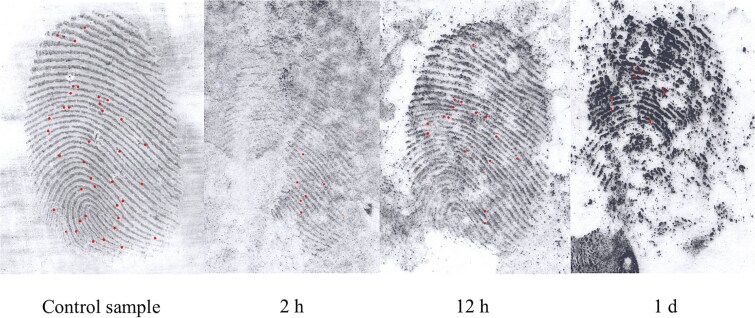
Degraded latent fingerprints affected by natural snowfall at different time intervals.

**Figure 3:**
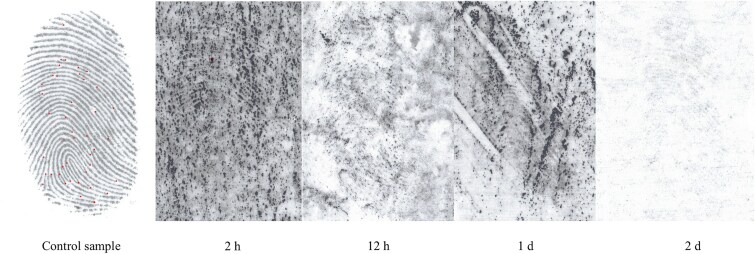
Degraded latent fingerprints due to forced snow immersion at different time intervals.

Considering that this is a pilot study, the method for evaluating latent fingerprints was developed by the author’s team. Previous studies, such as McCook et al. [22], examined the development of latent fingerprints after snow exposure but did not assess minutiae on degraded fingerprints. Therefore, we established the above procedure to evaluate latent fingerprints degraded by snow.

To determine the usability of fingerprints exposed to snowfall and forced immersion into the snow, they were categorized into three groups after development and evaluation, based on probability estimation and the experience of investigators in Slovakia, according to internal regulations [4]: usable (at least 10 minutiae), partially-usable (7–9 minutiae), and non-usable (fewer than seven minutiae).

The procedure of exposing fingerprints to snow environment under two different conditions with subsequent development and evaluation was carried out by a single experienced researcher in the field of forensic dactyloscopy.

Around 6 months after the first evaluation, 35 randomly selected latent fingerprints exposed to snow conditions were re-evaluated by the principal investigator to quantify intrapersonal error (agreement) using technical error of measurement (TEM).

### Statistical evaluation

Microsoft Office Excel 2023 was used for data processing, whilst the statistical analysis was carried out using SPSS version 26 (IBM Corp., Armonk, NY, USA).

Descriptive statistics of minutiae quantities on degraded latent prints exposed to snow and on forcibly immersed objects with latent prints in snow was assessed. Due to the non-normal distribution of the data, the non-parametric Kruskal-Wallis’s test was performed to test the differences in minutiae counts in relation to the different snow conditions and time intervals. The test was performed at the significance level *α* = 0.05. Values ˂0.05 were considered significant.

## Results

The TEM (0.059) and reliability coefficient (*R* = 0.98) indicate minimal measurement error in the data evaluation. This high reliability (where *R* ranges from 0 to1 and 1 presents no measurement error) confirms the precision of minutiae quantification on latent fingerprints.

### Latent fingerprint development success rate

After exposing latent fingerprints to snow conditions, we can conclude that it was possible to develop degraded latent fingerprints. Snowfall exposure produced higher-quality latent prints (35 developed per time interval, Figure 2) compared to forced snow immersion (40 developed per time interval, Figure 3).

### Effect of natural snowfall on latent fingerprints

Table 2 presents the descriptive statistics for the sample of degraded latent fingerprints resulting from natural snowfall, including details about the environmental factors observed. It includes the evaluated minutiae (min, max, mean±SD), the mean minutiae count as a percentage of the Control, the median, and the average ambient temperature. Natural snowfall reduced minutiae counts by: 73.20% (2 h), 60.31% (12 h), and 88.43% (1 d) *vs.* Control (all *P* < 0.001). Significant difference was found between 12 h *vs.* 1 d (*P* < 0.001), but not between 2 h *vs.* 12 h or 2 h *vs.* 1 d (*P* > 0.05).

**Table 2:** Descriptive statistics of the observed sample of average number of minutiae on degraded latent fingerprints affected by natural snowfall.

Sample	Number of fingerprints	Number of minutiae					Average temperature (°C)
		Min	Max	Mean ± SD	Mean % of Control	Median	
Control	35	26	45	38.80 ± 4.65	100.00	40	NA
2 h	35	0	36	10.40 ± 10.90^a^	26.80	9	−2.50
12 h	35	0	39	15.40 ± 13.47^a^	39.69	14	−2.25
1 d	35	0	30	4.49 ± 7.75 ^a,b^	11.57	0	−1.79

^a^ *P* < 0.001, compared with Control.

^b^ *P* < 0.001, compared with 12 h.

### Effect of forced snow immersion on latent fingerprints

Forced snow immersion reduced minutiae counts by: 98.21% (2 h), 99.59% (12 h), 100% (1 d), and 99.66% (2 d) *vs.* Control (all *P* < 0.05). No significant differences existed between any exposure time points (*P* > 0.05) (Table 3).

**Table 3:** Descriptive statistics of the observed sample of average number of minutiae on degraded latent fingerprints affected by forced snow immersion.

Sample	Number of fingerprints	Number of minutiae					Average temperature (°C)
		Min	Max	Mean ± SD	Mean % of Control	Median	
Control	40	35	53	43.58 ± 3.72	100.00	44.00	NA
2 h	40	0	10	0.78 ± 2.43^a^	1.78	0.00	0.50
12 h	40	0	5	0.18 ± 0.84^a^	0.41	0.00	−1.83
1 d	40	0	0	0.00 ± 0.00^a^	0.00	0.00	−1.92
2 d	40	0	4	0.15 ± 0.70^a^	0.34	0.00	−1.80

a *P* < 0.001, compared with Control.

### Usability of latent fingerprints after exposure to different snow conditions

Table 4 shows snow exposure significantly reduced fingerprint usability. Forced snow immersion caused the highest degradation (98.13% non-usable prints).

**Table 4:** Percentages of usable, partially usable, and non-usable fingerprints under two different conditions of snow factors.

Environmental factor	Number of fingerprints (*n*, %)				Average number of minutiae on fingerprints		
	Total	Usable	Partially usable	Non-usable	Usable	Partially usable	Non-usable
Natural snowfall	105	41 (39.05)	8 (7.62)	56 (53.33)	22	8	0
Forced snow immersion	160	0 (0.00)	3 (1.88)	157 (98.13)	0	8	0

## Discussion

This pilot study focuses on evaluating minutiae on degraded latent fingerprints due to snow conditions and then the usability of such degraded latent fingerprints. This topic has not been explored in previous research. In evaluating the average number of minutiae and the actual usability of fingerprints, we found that latent fingerprints that had been naturally snowed on had greater identification potential than those that had been forcibly immersed in the snow.

### Effect of natural snowfall on quality of latent fingerprints

In a study by Longchar et al*.* [31], they found that the best method to develop latent fingerprints exposed to snow conditions is a small particle agent. It is important to relocate latent fingerprints that have been exposed to snow conditions and temperatures of at least 0 °C to a room temperature environment before development. The SPR can only be applied to the surface once it is snow-free and the latent fingerprints have thawed [37]. In our work, we confirmed this observation after transferring latent fingerprints from a sub-zero environment to one above 0 °C. The main reason was to allow the snow to melt so that the latent fingerprints exposed to these conditions could be subsequently developed and evaluated.

We evaluated minutiae counts on groomed latent fingerprints of distal phalanges that were exposed to snow conditions. We also evaluated minutiae counts on control samples to which the degraded latent fingerprints were compared. Several differences were evaluated in the number of minutiae under the influence of each environmental factor. Differences in the usability of fingerprints that were degraded by the influence of selected environmental factors were also evaluated.

We confirmed the results of McCook et al*.* [22], in which they claimed that fingerprint quality may be decreased as a function of the time of exposure on latent fingerprints that had been naturally snowed on. Descriptive statistics show that the decrease in minutiae due to natural snowfall occurred at all sampling time intervals of degraded latent fingerprints compared to the control. There was an increase in the mean number of minutiae in the 12-h time interval compared to the 2-h and 1-d time intervals (Table 2). We assume that there is a fixation of the latent fingerprints and its components due to the influence of low temperature. The decrease in average minutiae count 1 d after snow exposure result from higher environment temperature during fingerprints deposition.

The quality of latent fingerprints also depends on the sweat-fat substance. During snowmelt, it may occur that the eccrine part of the sweat mixes with the melted snow. The eccrine component washes off more easily compared to the fatty component, which may decrease the quality of the latent fingerprints.

Based on the differences in minutiae count between the compared time intervals of natural snowfall exposure (Table 3), we can conclude that the time of exposure to snow has a negative effect on their quality (Figure 2).

McCook et al*.* [22] reported in their study that friction ridge skin was partially visible on latent fingerprints. We can confirm this statement because we were able to observe an image of the friction ridge skin after development with the SPR.

### Effect of forcible immersion into snow on quality of latent fingerprints

We can conclude that latent fingerprints exposed to forced snow immersion had lower visual quality than those exposed to natural snowfall. This may be influenced by the force that was exerted when they were immersed into the snow.

In the descriptive statistics, there is a significant reduction in the average number of minutiae of glass plates with latent fingerprints after only 2 h of exposure to forced snow immersion (Table 3), due to the mechanical removal of latent fingerprints when the glass plates were immersed in the snow. Another reason may be that the temperature of the environment was higher during the immersion of the object in the snow. This temperature gradually decreased afterwards. McCook et al*.* [22] hypothesized that there would be significant differences in the mean number of minutiae compared to the control. This would occur regardless of the amount of time the latent fingerprints were exposed forced snow immersion. Based on the results of the aforementioned study, we can confirm this hypothesis. The number of minutiae decreased to a minimum after the object was forcibly immersed in the snow.

The differences in the number of minutiae between the compared time intervals were due to a significant decrease in the average number of minutiae compared to the control sample. This decrease was observed as early as 2 h after the immersion of the object with latent fingerprints into the snow.

Also, the quality of developed fingerprints was lower. The mechanical force of the immersion of the object into the snow has the most significant influence on this destruction.

### Usability of latent fingerprints exposed to snow conditions

We observed significant differences after the influence of snow (Table 4). To confirm our findings, we evaluated two independent groups of latent fingerprint samples that were exposed to snow. In all categories of usable, partially-usable, and non-usable fingerprints, approximately the same number of fingerprints were evaluated in both observations. Latent fingerprints were left on glass plates in an environment with an average ambient temperature of −1.79 °C to −2.50 °C. Based on the results, it is clear that the length of exposure to snow has a negative effect on the latent fingerprints. After the latent fingerprints were developed, the minimum number of minutiae on most fingerprints were evaluated. An increased percentage of non-usable latent fingerprints were evaluated.

**Figure 4:**
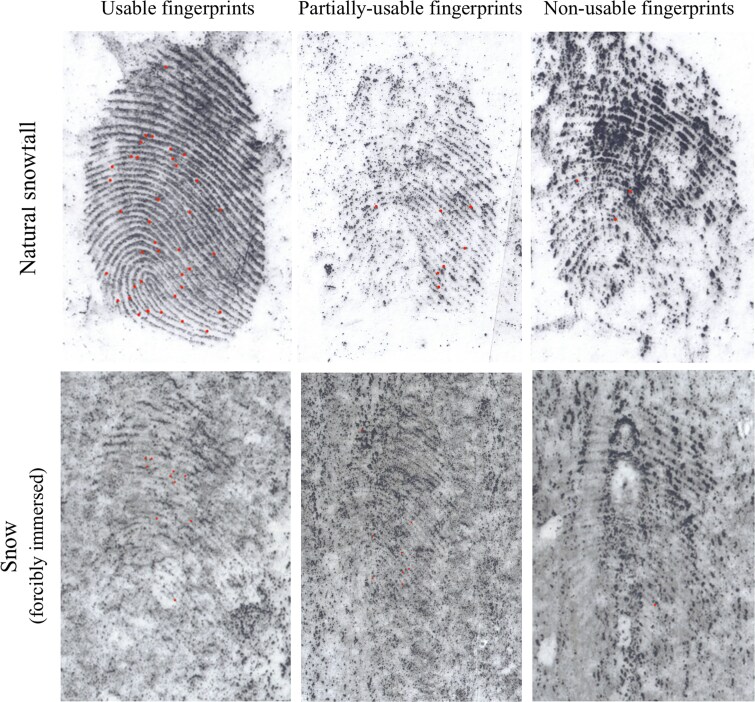
Comparison of usable, partially usable, and non-usable degraded latent fingerprints by each environmental factors.

The object that was forcibly placed in the snow was evaluated for the highest number of non-usable fingerprints. The percentage of non-usable fingerprints that were left on a non-porous surface and were subjected to forced immersion into the snow was 98.13%. These fingerprints would not be suitable for personal identification purposes. The reason for the large number of non-usable fingerprints is the mechanical removal of latent fingerprints. McCook et al*.* [22] evaluated that there were significant differences in an object forcibly embedded in snow on which latent fingerprints were left compared to a surface on which snow had fallen. We were able to confirm these results in our research (Figure 4), i.e., the observed fingerprints usability were consistent between natural snow exposure and forced snow immersion.

## Conclusion

Significant differences in the average number of minutiae were evaluated on fingerprints exposed to forced snow immersion. After exposing the latent fingerprints to this environmental factor, a decrease in the mean number of minutiae was evaluated by 98.21% after 2 h, 99.59% after 12 h, 100.00% after 1 d, and 99.66% after 2 d compared to the control sample. The most significant effect on these results was the mechanical removal of latent fingerprints after embedding the glass plates in snow. We can conclude that snow has a negative effect on the quality of latent fingerprints.

In conclusion, latent fingerprints exposed to natural snowfall have a greater identification potential with 39.05% of usable fingerprints than those exposed to forced snow immersion with 0.00% of usable fingerprints. We can also conclude that most of the non-usable fingerprints for personal identification were evaluated after the object was forcibly immersed into the snow.

## Study limitations and recommendations

Although the study is the first of its kind in Slovakia and Central Europe and makes an important contribution to the question of the identification potential of degraded latent fingerprints in relation to the external factor snow, there are some limitations and further recommendations that need to be mentioned. In the present study, the relative humidity of the environment, which may have influenced the results, was not considered and only the snow and selected time periods were evaluated as factors. Therefore, longer periods of the effect of snow under different conditions and other environmental factors, e.g., direct sunlight, soil environment, and others, should be further investigated. Since this study was conducted under uncontrolled conditions on the dactyloscopic glass plates, further studies could be conducted under controlled conditions and on different non-porous surfaces as suggested by McCook et al*.* [22] and Dhall and Kapoor [30].
